# PNT2258, a novel deoxyribonucleic acid inhibitor, induces cell cycle arrest and apoptosis via a distinct mechanism of action: a new class of drug for non-Hodgkin's lymphoma

**DOI:** 10.18632/oncotarget.9872

**Published:** 2016-06-07

**Authors:** Abdul Shukkur Ebrahim, Mustapha Kandouz, Allison Liddane, Hussam Sabbagh, Yuning Hou, Chunying Li, Ayad Al-Katib

**Affiliations:** ^1^ Lymphoma Research Lab, Wayne State University, Detroit, MI, 48201-USA; ^2^ Department of Pathology, Wayne State University, Detroit, MI, 48201-USA; ^3^ Department of Biochemistry and Molecular Biology, Wayne State University, Detroit, MI, 48201-USA

**Keywords:** non-Hodgkin's lymphoma, WSU-FSCCL, PNT2258, DNAi, apoptosis

## Abstract

Current therapy for BCL-2-associated tumors such as Non-Hodgkin Lymphomas (NHL) is inadequate. The DNAi PNT2258, a 24 base single-stranded phosphodiester DNA oligodeoxynucleotide (PNT100) encapsulated in a protective liposome, was precisely designed to treat cancers that over-express *BCL-2.* PNT2258 strongly inhibited BCL-2 promoter activity, confirming its predicted mechanism of action. BCL-2 mRNA and protein expression were significantly downregulated in a follicular small cleaved cell lymphoma (WSU-FSCCL) cell line. 2.5μM PNT2258 induced an initial S- phase arrest followed by a gradual increase in the sub-G0 (apoptosis) compartment and a reciprocal progressive decrease of the S phase. Terminal deoxynucleotidyl transferase (TdT)-positive populations and cleaved caspase-3 and PARP were also increased. The data are consistent with the idea that BCL-2 inhibition by PNT2258 activates apoptotic pathways in WSU-FSCCL cells. This is the first report to address the distinct mechanism of action underlying the anti-BCL-2 functions of PNT2258. Growth inhibition in two other cell lines, WSU-DLCL2 and WSU-WM, supports broad applicability of BCL-2 DNAi to treatment of B-cell NHL.

## INTRODUCTION

Non-Hodgkin's lymphoma (NHL) is the 7^th^ most common cancer in the US [1], with 530,919 reported cases [2]. 71,850 new cases (39,850 males and 32,000 females) were predicted for 2015 [3]. NHL is a group of heterogeneous diseases resulting from malignant transformation of lymphoid cells with different molecular pathogeneses [4]. 90% of NHLs involve B cells; of these, 30% are characterized as aggressive and 20% as indolent [5]. Diffuse large B-cell lymphoma (DLBCL), representative of aggressive NHLs, is often curable but follicular lymphoma (FL), the prototype indolent NHL, remains difficult to treat [6, 7].

BCL-2 (B-cell lymphoma 2), a key regulator of apoptosis first cloned from a human B-cell lymphoma, is expressed in a number of hematologic malignancies and solid tumors. BCL-2 is overexpressed in both FL and DLBCL, mostly as a result of a t(14;18) translocation [8–10]. The translocation, a cytogenetic hallmark of FL, is seen in 80%-90% of cases [11, 12] and in 30% of cases of DLBCL [13, 14]. 10% of FL tumors lack the translocation and do not express BCL-2 protein: this is common in grade 3b, which is dominated by centroblasts [15, 16].

To date, most methods aimed at interfering with the anti-apoptotic effects of BCL-2 have relied on inhibition via small molecules such as ABT-199 or on decreasing expression at the RNA level via RNAi [17–26]. In this article, we describe a novel approach, the use of a specific oligonucleotide to block transcription by DNA interference (DNAi) [27].

We recently reported on the development, physiochemical characterization and activity of the DNAi, PNT2258 [28]. PNT2258, a 24 base-single stranded, unmodified phosphodiester DNA oligonucleotide (PNT100) encapsulated in specialized liposomes (SMARTICLES^®^), was designed to target an untranslated region of *BCL-2* to block transcription. The drug exhibited broad antitumor activity *in vitro* and in xenograft models [28]. PNT2258 was shown to be safe and well-tolerated at doses of up to150 mg/m^2^ in a phase I study [29] and to have activity in patients with relapsed or refractory NHL in a pilot phase II study [30]. We hypothesized that PNT2258 inhibits *BCL-2* via direct inhibition of transcription, although it may affect promoter or transcription factor binding. *BCL-2* contributes to the genesis of lymphomas, is critical for cancer cell survival, and promotes chemo-resistance [31]. Current chemotherapeutic treatment options are non-specific and cause significant off-target toxicity. Specific targeting of the BCL-2 family of proteins offers the opportunity to minimize off-target effects and control apoptotic and survival pathways directly.

In the current study, we used three lymphoma cell lines with distinct genetic characteristics, WSU-FSCCL (follicular small cleaved cell lymphoma; t(14;18) *BCL-2* and t(8;11) *c-myc* rearrangements), WSU-DLCL2 (diffuse large cell lymphoma; t(14;18) *BCL-2* rearrangement) and WSU-WM (Waldenstrom's macroglobulinemia; t(8;14) *c-MYC* rearrangement), to investigate the mechanism of action of PNT2258 and its role as a DNAi. Our results show that the down regulation of BCL-2 mRNA and protein expression following PNT2258 exposure triggers cell death pathways in FSCCL cells. We conclude that DNAi is a novel gene-silencing strategy that could be applied to therapeutic targeting of a variety of genes important to different types of cancer.

## RESULTS

### PNT2258 represses the BCL-2 promoter

**Figure 1:**
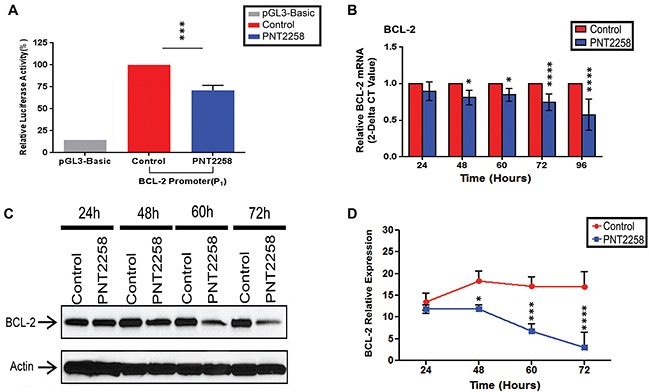
PNT2258 represses BCL-2 promoter activity and down regulates BCL-2 protein and mRNA expression **A.** Luciferase activity of K562 cells transiently transfected with the *BCL-2* promoter construct was significantly lower in the presence of PNT2258. Results are representative of three independent experiments (***P<0.001 by ANOVA). **B-D.** WSU-FSCCL cells were treatedwith 2.5μM PNT2258 for the indicatedtimes. (B) Quantitative RT-PCR analysis indicates that PNT2258 inhibition of BCL-2 occurs at the level of transcription. *RPLPO* was used as control. (C) Representative Western blots demonstrating time-dependent decreases in BCL-2 protein in PNT2258-treated cells; β-actin was used as loading control. (D) Densitometric analysis of BCL-2 band intensities (24 h-72 h) normalized to β-actin. Results are representative of three independent experiments. (* P<0.05, ***P<0.001, and ****P<0.0001 by ANOVA for B and D).

The DNAi sequences used here, complementary to the template strand of the DNA, were originally screened against the ENCODE database to ensure that the targeted areas did not encode mRNA or microRNA. The 25 mer PNT100 sequence of PNT2258 is complementary to a segment of the consensus sequence recognized by the Sp1 transcription factor in the *BCL-2* P1 promoter. We first sought to determine whether a putative 2638-bp (−3934 to-1287) region in the *BCL-2* promoter (P1) confers PNT2258 responsiveness by transiently transfecting K562 cells with a plasmid containing the *BCL-2* promoter construct before treatment with or without PNT2258 for 48 h. As shown in Figure 1A, PNT2258 strongly down-regulated *BCL-2* promoter activity.

### PNT2258 down-regulates BCL-2 protein and mRNA expression in WSU-FSCCL cells

We next examined whether PNT2258 inhibits BCL-2 expression in WSU-FSCCL cells. In this study, we compared PNT2258-treated and non-treated cells as we previously reported that three PNT2258 control sequences (scrambled, mismatched and reverse complement) had no anti-proliferative effects [28]. RT-PCR (Figure 1B) and Western blot (Figure 1C, 1D) analysis revealed that BCL-2 mRNA and protein levels were significantly decreased from 48 h through 72 h after exposure to 2.5μM PNT2258. Since BCL-2 is known to prevent apoptosis, we investigated whether PNT2258 treatment induced apoptosis in these cells and in two other lymphoma cell lines with different BCL-2 expression characteristics.

### PNT2258 decreases lymphoma cell viability

**Figure 2:**
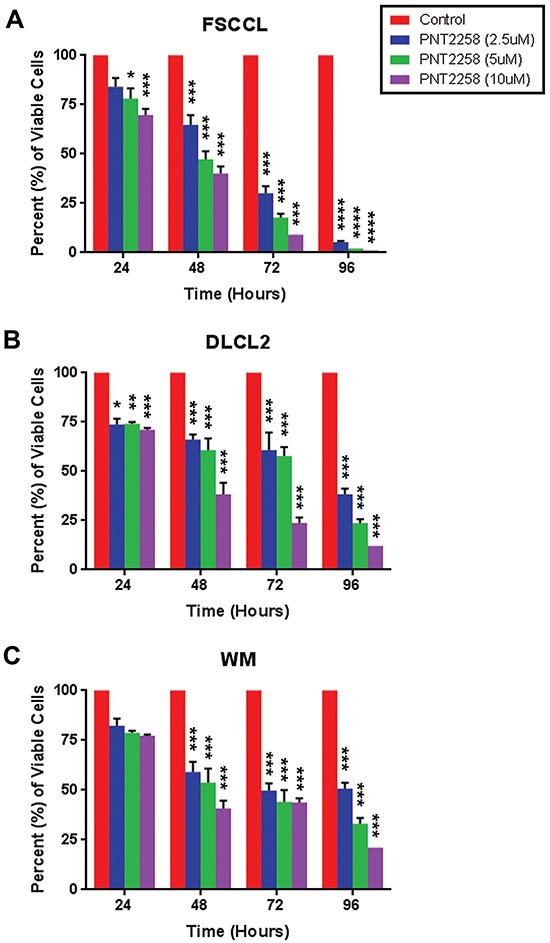
*In vitro* dose- and time-dependent lymphoma cell growth inhibition by PNT2258 Cell viability was measured by 0.4% trypan blue exclusion every 24 h over 96 h in **A.** WSU-FSCCL, **B.** WSU-DLCL2, and **C.** WSU-WM lymphoma cell lines exposed to PNT2258 (2.5μM, 5μM, and 10μM) from time 0. PNT2258 decreased cell viability in all cell lines but was most effective in WSU-FSCCL cells. All values represent mean ± SE of triplicate experiments. (* P<0.05, **P<0.01, ***P<0.001, and ****P<0.0001 by ANOVA).

We treated three lymphoma cell lines, two with t (14;18) and BCL-2 rearrangements (WSU-FSCCL and WSU-DLCL2) and one (WSU-WM) lacking these rearrangements, with different concentrations (2.5μM, 5.0μM, and 10μM) of PNT2258. Each of the cell lines expressed BCL-2 at baseline (Supplementary Figure S1). Cell viability was decreased in a dose dependent manner: growth inhibition between 2.5μM and 10 μM was statistically significant in all three cell lines at 48h-96h, but differences between 2.5 μM and 5 μM and 5 μM and 10 μM were variable. Cell viability was significantly lower in WSU-FSCCL cells at 24h. As predicted, NHL cell lines with the (14;18) translocation were more sensitive to PNT2258 than the cell line without these alterations (Figure 2A–2C). We chose to continue our investigations in the cell line with the greatest response, WSU-FSCCL (Figure 2A; *p* ≤ 0.0001).

### PNT2258 induces cell cycle arrest and apoptosis

**Figure 3:**
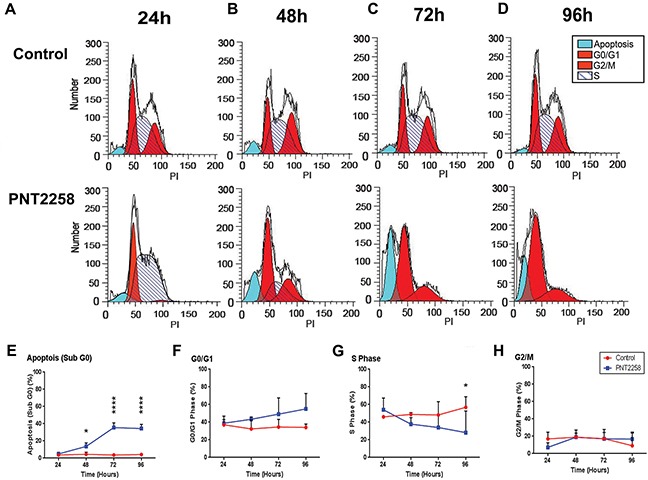
PNT2258 induces S phase cell cycle arrest in WSU-FSCCL cells FSCCL cells weretreated for 24-96 h with 2.5μM PNT2258and hypotonically lysed in propidium iodide. Nuclei were analyzed for DNA content by flow cytometry. **A-D.** Original flow data for each time point. **E-H.** Percent of cells in each phase of the cell cycle (sub-G0, G0/G1, S, G2/M) at each time point. All values represent mean ± SE of triplicate experiments. (* P<0.05, **P<0.01, ***P<0.001, and ****P<0.0001 by ANOVA).

To determine whether PNT2258 induced cell death through on-target intrinsic apoptosis, WSU-FSCCL cells exposed to 2.5μM PNT2258 for up to 96 h were analyzed by flow cytometry. The S-phase population was increased (from 46% to 54%; Figure 3A and 3G) and the proportion of cells in G2-M was reduced (from 16% to 7%; Figure 3A and 3H) at 24 h. There was a reciprocal increase in the sub-G0 compartment (apoptosis) and G0/G1 (resting cells) starting at 48 h (Figure 3B and 3E) with a progressive increase in sub-G0 (from 3% to 35%; Figure 3C and 3E) and G0/G1 (from 34% to 55%; Figure 3C, 3D and 3F) at 72 and 96 h. There was a significant decrease in S-phase cells (from 56% to 28%; Figure 3D and 3G) at 96 h compared with control. The results are summarized in Supplementary Table S1. PNT2258 induces: 1) apoptosis, and 2) cell cycle arrest at G0/G1 (growth inhibition), indicating an effect on the G0/G1 checkpoint. Since current anti-BCL-2 drugs usually cause apoptosis without affecting the cell cycle, our results suggest that PNT2258 may control the cell cycle via non-BCL-2 mechanisms.

**Figure 4:**
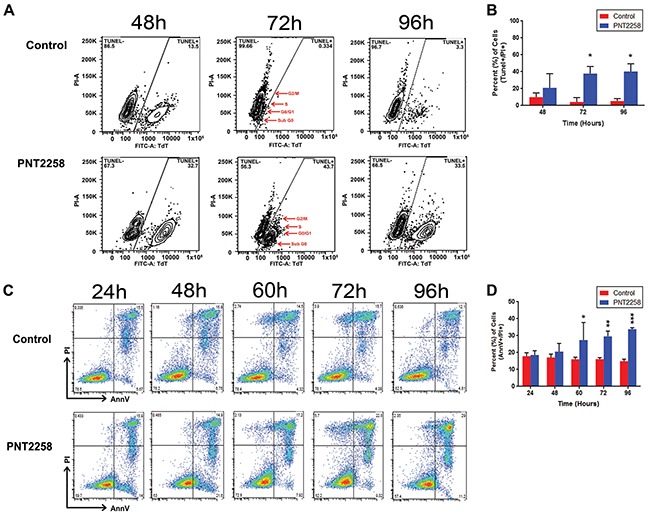
PNT2258 induces apoptosis WSU-FSCCL cells, treated with 2.5 μM PNT2258 for 24-96 h, were analyzed for terminal transferase dUTP Nick End Labeling (TUNEL) staining by flow cytometry. **A.** Flow data of FITC positive cells by cell cycle phase (sub G0, G0/G1, S, G2/M). The largest FITC positive population in PNT2258-treated cells was in G0/G1 followed by sub-G0 (red arrows) at 72-96 h. **B.** Percent of apoptotic cells was significantly higher in PNT2258 treated cells (* P<0.05 by ANOVA). **C.** Complete time course (24 h −72 h) flow cytometric analysis of apoptosis in cells stained with Annexin V-FITC. **D.** PNT2258 increased the percent of apoptotic cells from 60- 96 h (* P<0.05, **P<0.01, and ***P<0.001 by ANOVA). Results are representative of three independent experiments.

Further examination of the PNT2258 effect on apoptotic cell death in WSU-FSCCL demonstrated that, at the lowest concentration used (2.5uM), PNT2258 elicited the formation of the distinct morphologic features of apoptosis, including cell shrinkage, nuclear chromatin condensation, and formation of membrane blebs and pyknotic bodies after 72h of treatment (data not shown). TUNEL assays show an increase in the FITC-TdT-positive population in PNT2258 treated cells compared with control (horizontal axis) (Figure 4A). Total FITC positive cells also increased with increasing time of incubation, from 20% at 48h to 40% at 72h (Figure 4B). Moreover, there was a progressive shift-to-the-right of the FITC positive cell population with increasing incubation time indicating increasing frequency of DNA breaks (apoptosis). Most of the FITC positive cells were in the sub-G0 phase; however, there were also positive cells in G0/G1, S and G2/M. Induction of apoptosis was confirmed independently using AnnexinV staining as shown in Figure 4C and 4D.

### PNT2258 increases cleaved caspase-3, and cleaved PARP and decreases Mcl-1 and cytochrome C protein levels

**Figure 5:**
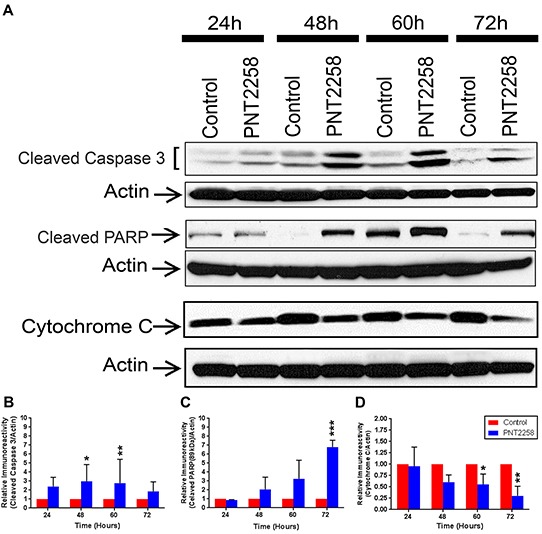
Cleavage of Caspase 3 and PARP protein and down regulation of Cytochrome C in PNT2258 treated WSU-FSSCL cells **A.** Representative Western blots of cleaved Caspase 3 and cleaved PARP and Cytochrome C in WSU-FSCCL cells incubated with 2.5μM PNT2258 for 24-72 h; β-actin was used as loading control. **B-D.** Densitometric analysis indicates that cleaved Caspase 3 and PARP levels were significantly higher (B and C) and Cytochrome C levels were lower (D) in PNT2258 treated cells than in the control. Band intensities were normalized to β-actin. Results are representative of three independent experiments. (* P<0.05 and **P<0.01 by ANOVA for B, C and D).

We then assessed expression of several BCL-2 family members and selected markers of apoptosis in our PNT2258-treated WSU-FSCCL cells. Protein levels of Mcl-1L and Mcl-1S were decreased (Supplementary Figure S2A, S2B, S2C) but expression of Bcl-xL, Bax, Bak, Bid, and p53, remained unchanged (Supplementary Figure S2A and S2C). However, there was activation of the apoptosis executioner (caspase-3) and induction of both caspase-3 and PARP cleavage (Figure 5A, 5B, 5C) as well as a reduction of Cytochrome C (Figure 5A and 5D), supporting flow cytometry data (Figures 3 and 4) that 2.5μM PNT2258 induces significant apoptosis.

### BCL-2 overexpression represses PNT2258-induced cell death

To document that PNT2258 induces cell death via downregulation of BCL-2, K562 cells were transfected with a pcDNA3 expression vector containing BCL-2 cDNA or the control empty vector before treatment with PNT228. Cell viability, assessed by Trypan blue, was significantly lower in PNT2258-treated cells transfected with the empty vector at 96 hr but not in those expressing BCL-2 (Supplementary Figure S3).

## DISCUSSION

DNA interference (DNAi) sequences are designed to target regions upstream of the ATG start site of genes that cause disease. These regions lie within CG-rich areas and, by design, coincide with (or are near) consensus motifs recognized by transcription factors or other cis-regulatory acting elements. Gene transcription is inhibited when sufficient DNAi molecules are available. The PNT100 sequence used for this study specifically targets a region recognized to be regulatory for *BCL-2* that is translocated with (14;18) chromosomal rearrangement [28]. The results presented above indicate that PNT2258, a prototype DNAi, is most effective at decreasing cell viability in BCL-2 expressing lymphoma cell lines with the t(14;18) translocation. PNT2258 induced cell cycle arrest and increased apoptosis. BCL-2 expression was significantly inhibited at both the mRNA and protein levels, suggesting that this decrease in BCL-2 expression is responsible for PNT2258-induced apoptosis.

It is noteworthy that PNT2258 induced its therapeutic effects on lymphoma cells at concentrations achievable in patients. A 2.5 μM concentration of PNT2258 translates to 17.6 μg/mL (mol. weight of PNT2258 is 7221g/mol). According to the pharmacokinetic analysis conducted as part of the phase I clinical trial, a maximum plasma concentration of 29.222 μg/mL (cycle 1 day 1) and 42.125 μg/mL (cycle 1 day5) were achieved at a dose of 113 mg/ m^2^ [30]. The selected safe dose utilized in phase II trial was 120mg/m^2^.

BCL-2 is a logical target for the treatment of NHL and has been the focus of significant drug development efforts. Our group has developed and evaluated different anti-BCL-2 strategies including small molecule inhibitors (SMIs) [20, 42–45] and anti-sense oligonucleotides [23, 27]. RNAi often requires large doses and continuous exposure for effective suppression of gene function. SMI toxicity varies by type: pan BCL-2 homology domain 3 (BH3) mimetics (AT-101) tend to be more toxic, causing dose-dependent thrombocytopenia [17, 46, 47], selective BCL-2 BH3 mimetics, although platelet sparing, induce resistance and increase the risk of tumor lysis syndrome [17, 48, 49]. Side effects and host toxicity may limit the ability to deliver effective dosages of all these treatment regimens. These limitations can be overcome if one can target a gene of interest at the DNA level.

PNT2258 has shown great promise in preclinical and pilot phase II clinical evaluations (28, 30). The DNAi was designed to target the non-coding, non-transcribed regions 5′ upstream of the ATG start site in the *BCL-2* promoter. Disruption of Sp1 results in transcriptional inhibition of BCL-2 [50]. Sp1 binds to double-stranded DNA and recognizes the consensus sequence 5′- G/T (GGGCGG) G/AA -3′, and, coincidentally, a complementary sequence within PNT100 (28). The DNAi sequence used here is unique and 100% homologous to the CpG region of BCL-2. Off-target binding to other Sp1 sites, and/or genomic DNA or RNA is unlikely. Our results confirm that the region between 1506 bp and 1737 bp of the *BCL-2* promoter confers PNT2258 responsiveness. BCL-2 overexpression protected K562 cells from PNT2258-induced cell death. The fact that BCL-2 mRNA and protein expression were also inhibited support a DNAi mechanism of action.

The mechanisms of action of this new DNAi are just beginning to be understood [51]. Induction of apoptosis by PNT2258 in WSU-FSCCL cells is predominantly the result of its actions on BCL-2 expression. Its additional effects on cell cycle arrest suggest PNT2258 may have secondary targets as BCL-2 is not involved in cell cycle arrest. It is conceivable that DNAi oligonucleotides may bind to other BCL-2 family proteins. In our hands, only Mcl-1 protein levels were lower in PNT2258-treated cells. However, we believe that this decrease is secondary to the cell cycle changes induced by PNT2258 as Mcl-1 down-regulation was recently shown to be associated with apoptosis and G_1_/S transition [52]. The possibility that PNT2258 may target both intended (BCL-2) and unintended genes, thus enhancing its effectiveness, remains to be investigated further.

Although some lymphomas can be competently treated with a combination of chemotherapy and immunotherapy, many patients eventually become refractory to these treatments and ultimately succumb to their disease. To our knowledge, this is the first systematic study showing the selective induction of apoptosis and cell cycle arrest in lymphoma cells treated with PNT2258, suggesting potential translational application of PNT2258 as a safe treatment for NHL, including recurrent disease. DNAi oligonucleotides may prove to be an essential tool for future therapeutic intervention, particularly in cancer.

## MATERIALS AND METHODS

### Cell culture

The cell lines used in this study (WSU-FSCCL, WSU-DLCL2, and WSU-WM), established at Wayne State University (WSU) [32–34] and EBV (Epstein-Barr virus) negative were cultured as previously described [35]. Molecular characterization, translocations and breakpoints, of each cell line have been published [32]. Human K562 cells were maintained according to a standard protocol (ATCC, Manassas, VA).

### Transient transfections and luciferase assays

Constructs containing the P1 promoter (LB124: −3934 to −1287) in Bluescript were purchased from Addgene (Cambridge, MA). The P1 promoter construct was obtained by digesting LB124 with SpeI and HindIII and inserting the P1 promoter region into NheI (which has SpeI-compatible overhangs)/HindIII restricted pGL3-Basic (Promega, Madison, WI) that contains the reporter firefly luciferase gene. All transfections were carried out in 24 well-plates, Thermo Scientific, (Rockford, IL, USA). Briefly, plasmids (1 μg *BCL-2* promoter construct, 0.1 μg Renilla luciferase-expressing reporter vector pRL-Null (Promega; Madison, WI), were introduced into < 50% confluent cells with Lipofectamine^®^ LTX transfection reagent (Life Technologies; Grand Island, NY). Cells were lysed 48 h later, and promoter activity analyzed in a MicroLumat Plus LB96V (Berthold Technologies) using the Dual Luciferase^®^ Reporter Assay System (Promega). The firefly luciferase values were normalized to those of Renilla luciferase; all transfections were repeated at least three times.

### Antibodies and reagents

The following antibodies were obtained from Cell Signaling Technology (Danvers, MA, USA): BCL-2 (2876, 1:1000), Caspase-3 (9662, 1:1000) and PARP (9532, 1:1000). The following antibodies were purchased from Santa Cruz Biotechnology (Santa Cruz, CA, USA): Cytochrome C (7H8: sc-13560, 1:1000), Bcl-xL (H-5: sc-8392, 1:500), Mcl-1 (S-19: sc-819, 1:1000), Bax (N-20: sc-493, 1:500), Bak (G-23: sc-832, 1:500), Bid (FL-195: sc-11423, 1:1000), p53 (DO-1: sc-126, 1:500), and the actin antibody (ACTN05, 1:2000) from Thermo Scientific, (Rockford, IL, USA). Protein concentrations were determined using the Micro bicinchoninic acid (BCA) protein assay (Pierce Chemical Company, Rockford, IL, USA). PNT2258 and associated reagents were provided by ProNAi Therapeutics (Ann Arbor, MI).

### Immunoblotting

PNT2258-treated and untreated cells were harvested, washed in PBS and lysed in M-PER lysis buffer containing a protease and phosphatase inhibitor cocktail (Thermo Scientific, Rockford, IL, USA) [36]. Equal amounts of protein lysates were subjected to SDS-PAGE followed by blotting with the indicated antibodies and detection by Western Super Signal West Pico Chemiluminescent substrate reagents (Thermo Scientific, Rockford, IL, USA). Select images were quantified using ImageJ densitometry software (Version 1.45, US National Institutes of Health) and normalized to the actin signal. Data are presented as relative band signal intensity compared to control.

### Quantitative real-time PCR analysis

RNA was extracted from PNT2258 treated and untreated control lymphoma cells using the miRNeasy Micro Kit (Qiagen, Valencia, CA, USA). Total RNA was quantified by NanoDrop and 1μg of each sample was reverse-transcribed using the SuperScriptW VILOTM cDNA synthesis kit according to the manufacturer's instructions (Life Technologies, Invitrogen). The *BCL-2* and *RPLPO* genes were used for detection of gene amplification and normalization of each sample. Reverse transcription and PCR were performed using the qRT-PCR Primer Set (Life Technologies, Invitrogen). PCR product amplification was detected by the level of fluorescence emitted by SYBR Green I master (Roche, Pleasanton, CA, USA) according to manufacturer's protocol. Reactions were carried out in a 384 well microtiter plate using the LightCyclerW 480 System (Roche). Three independent experiments were performed in triplicate and each reaction was repeated at least once to ensure accuracy. The PCR cycle number at threshold (Ct) was used for the comparison. Gene expression at baseline and post treatment was quantified by qRT-PCR relative to RPLPO using the ΔΔCt method and expressed as fold change of gene expression relative to that in untreated control [37]. Values represent mean ± SE of three independent experiments performed in triplicate.

### Growth inhibition by trypan blue

Treated and control cells were seeded at a density of 0.2×10^6^ viable cells/ml per well in a 24-well plate (Costar, Cambridge, MA, USA) and treated with PNT2258 (at 2.5, 5, and 10 μM) for variable periods of time. The number of viable cells was determined by trypan blue exclusion (Sigma Chemical Co. St. Louis, MO, USA) at 24 hour intervals [38].

### Cell cycle analysis

PNT2258-treated and control cells were collected and centrifuged twice in cold PBS. Cells were then fixed in 5 ml of 70% ethanol and stored at 4°C overnight [38, 39]. For analysis, cells were centrifuged and resuspended in 1 ml of staining buffer containing 50ug/ml propidium iodide, 100ug/ml of RNase A, and 0.1% of Triton X-100. The DNA content was then analyzed on a Coulter EPICS 753 flow cytometer and the different stages of the cell cycle were determined using a ModFit 5.2 computer program. The flow cytometry work was done at the Microscopy, Imaging and Cytometry Resources Core at Wayne State University.

### Apoptosis assay

Apoptotic cell death was determined using Annexin V-FITC staining and TUNEL assay (Bio-Vision Mountain View, CA). The Annexin V-FITC studies were performed according to the manufacturer's instructions but the protocol for ApoDIRECT (TUNEL assay) was slightly modified as previously published by our lab [38]. In brief, the fluorescein-labeled DNA was detected by flow cytometry. PI staining was simultaneously used to separate cells into G0/G1, S, G2/ M, and sub-G0 compartments based on DNA content. Since dUTP-positive cells are considered apoptotic, dual staining with dUTP and PI allowed us to assign dUTP-positive cells to a specific phase of the cell cycle [40]. The percentage of apoptotic cells was determined by flow cytometric analysis of the sub-G0/G1 DNA population in cell-cycle histograms as described in detail elsewhere [38].

### Transfections and rescue experiment

The BCL-2 expression vector used in the study has been previously described [41]. The BCL-2 and control empty vectors (pcDNA3) were transfected into K562 cells at a density of 0.2×10^6^ viable cells/ml per well in a 24-well plate. 24hrs after transfection, cells were treated with 10 μM PNT2258 for variable periods of time. The number of viable cells was determined by trypan blue exclusion (Sigma Chemical Co. St. Louis, MO, USA) at 24 hour intervals [38].

### Data analysis and statistical significance

Statistical analyses were performed by one-way ANOVA using PRISM. ImageJ densitometry software (Version 1.45, US National Institutes of Health) was used for quantification of Western blot bands. Selected bands were quantified based on their relative integrated intensities, calculated as the product of the selected pixel area and the mean gray value for those pixels normalized to the internal control. Fold increase or decrease was calculated by standardizing each treatment as a ratio to the control. Statistical significance was set at p < 0.05 for all data comparisons.

## SUPPLEMENTARY FIGURES AND TABLE




