# Supplementary material for: PNT2258, a novel deoxyribonucleic acid inhibitor, induces cell cycle arrest and apoptosis via a distinct mechanism of action: a new class of drug for non-Hodgkin's lymphoma
Source: Oncotarget. 2016 Jun 7;7(27):42374–84. doi: 10.18632/oncotarget.9872 (PMC5173141; doi:10.18632/oncotarget.9872)
Supplement: Supplementary file 1 [file oncotarget-07-42374-s001.pdf]

SUPPLEMENTARY FIGURES AND TABLE

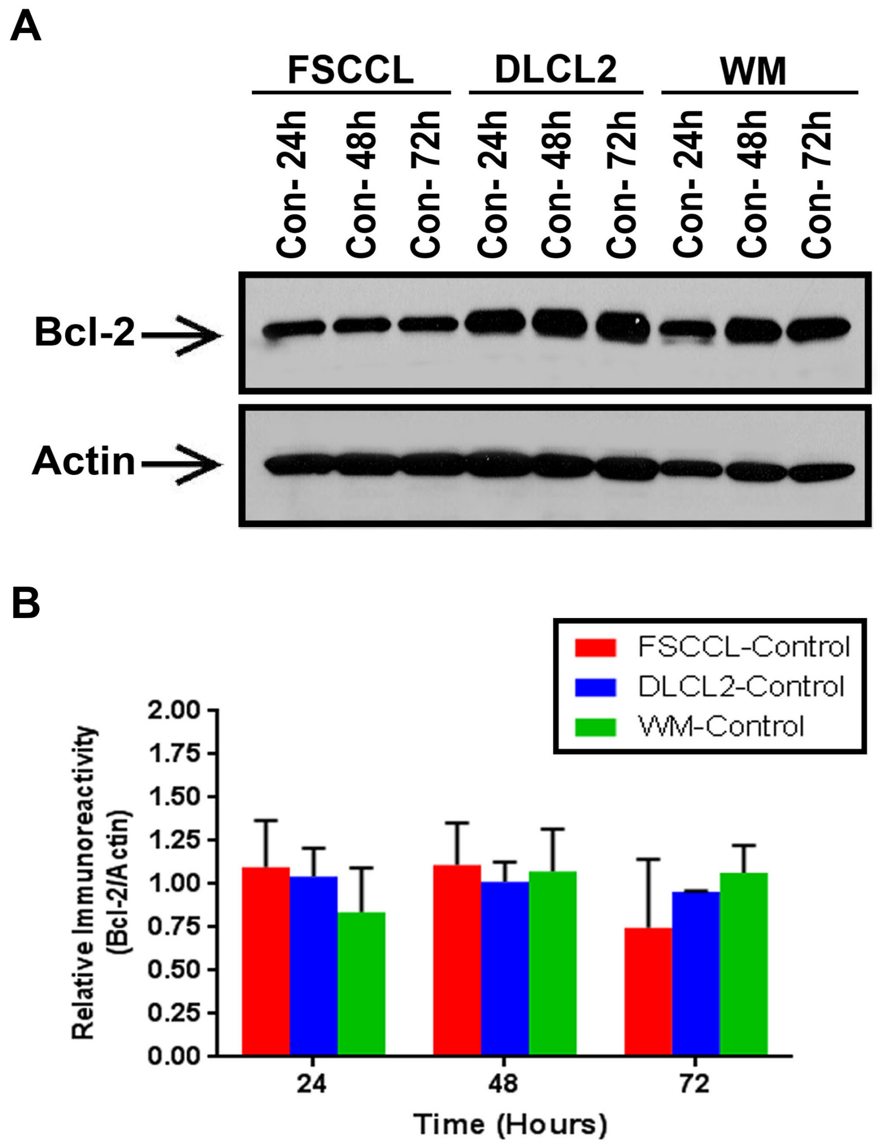

Supplementary Figure S1: A. Western blots demonstrating time-dependent BCL-2 expression in WSU-FSCCL, WSU-DLCL2 and WSU-WM cells; β-actin was used as loading control. **B.** Densitometric analysis of BCL-2 band intensities (24 h-72 h) normalized to β-actin.

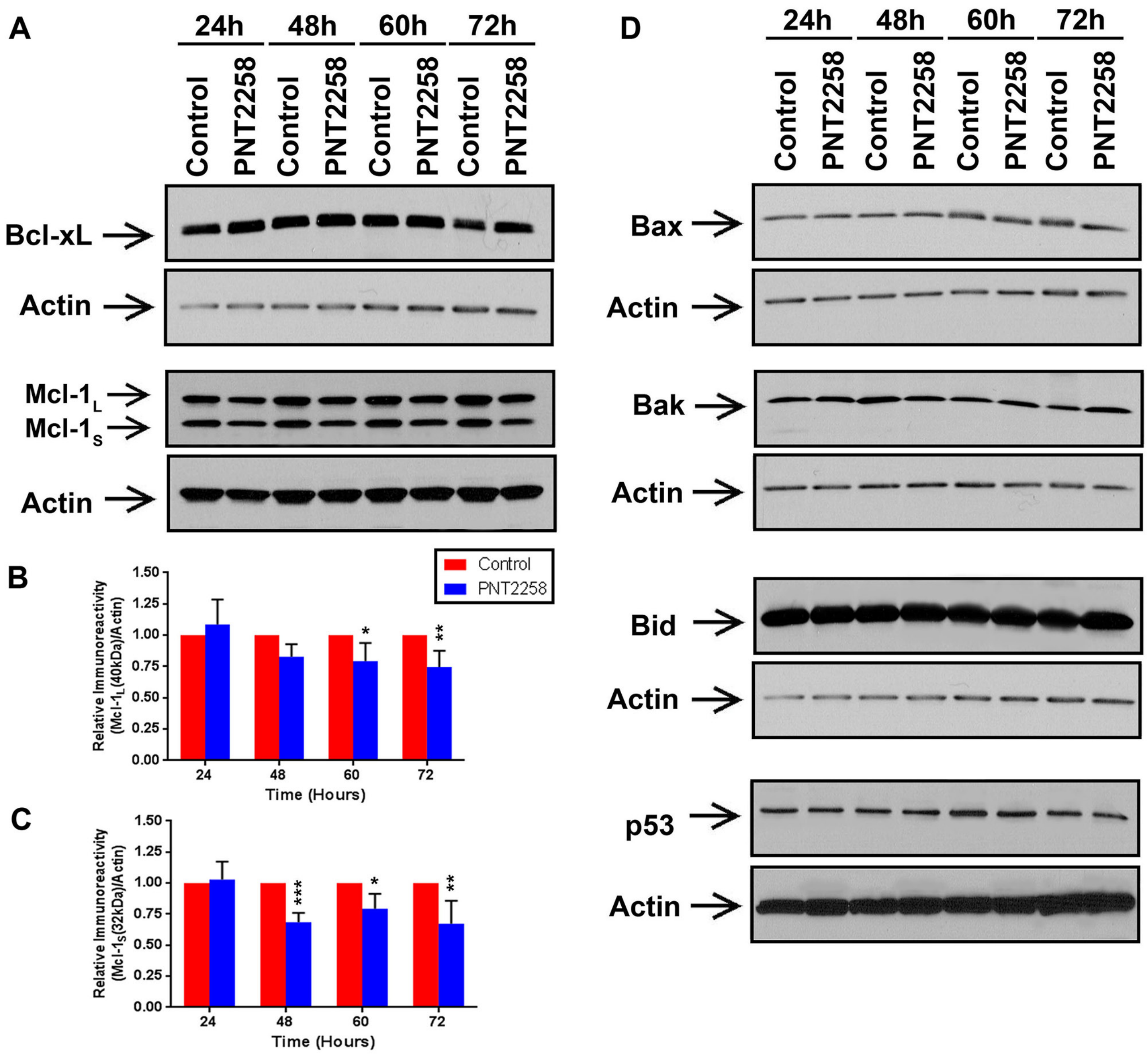

Supplementary Figure S2: A. Representative Western blots of Bcl-xL, Mcl-1_L and Mcl-1_S in WSU-FSCCL cells incubated with 2.5μM PNT2258 for 24-72 h; β-actin was used as loading control. **B-C.** Mcl-1_L and Mcl-1_S levels were significantly lower in PNT2258-treated cells. Densitometric analysis of band intensities normalized to β-actin. **D.** Bax, Bak, Bid and p53 levels were unchanged by PNT2258 treatment. Results are representative of three independent experiments. (* P<0.05, **P<0.01 and ***P<0.001 by ANOVA for B and C).

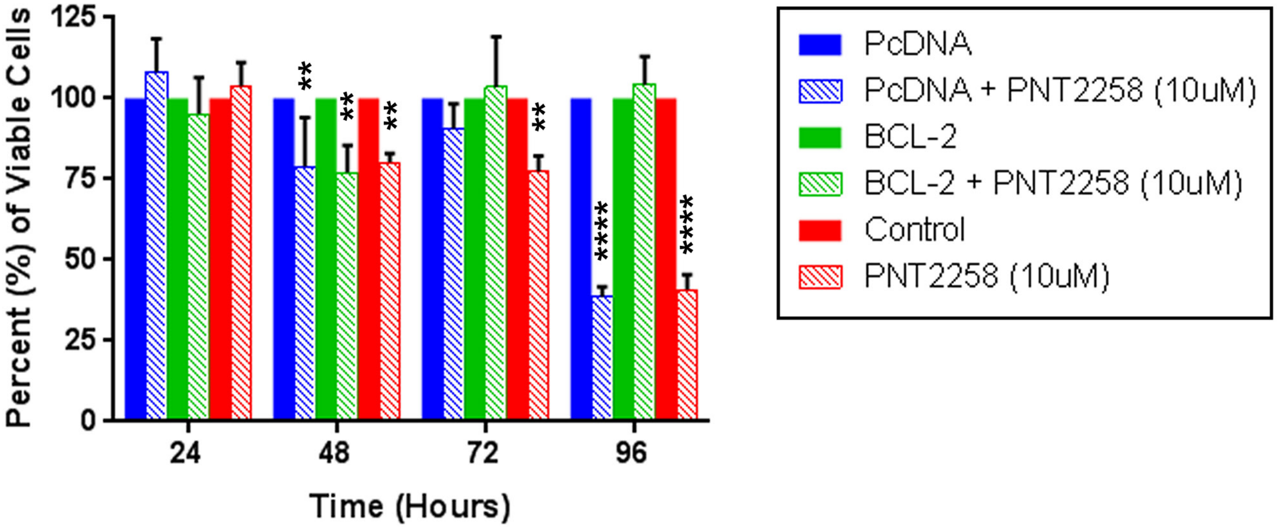

Supplementary Figure S3: After transfection with BCL-2 or pcDNA3 vectors, K562 cells were continuously cultured for 4 days in the presence or absence of 10 µM PNT2258. At different treatment time, cells were taken and analyzed for cell death by Trypan blue assays. Results are representative of three independent experiments. (* $P<0.05$, ** $P<0.01$ and *** $P<0.001$ by ANOVA).

Supplementary Table S1: Table demonstrating flowcytometry data.

See Supplementary File 1
