# Supplementary figures and images for: PNT2258, a novel deoxyribonucleic acid inhibitor, induces cell cycle arrest and apoptosis via a distinct mechanism of action: a new class of drug for non-Hodgkin's lymphoma
Source: Oncotarget. 2016 Jun 7;7(27):42374–84. doi: 10.18632/oncotarget.9872 (PMC5173141; doi:10.18632/oncotarget.9872)

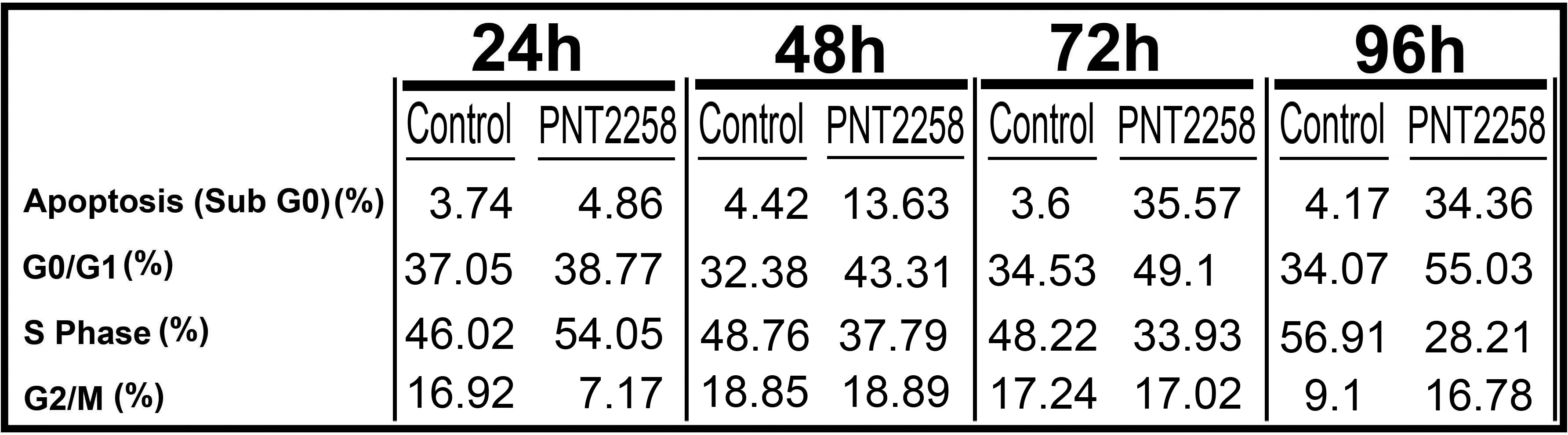

Supplement: Supplementary file 2 [file oncotarget-07-42374-s002.tif]
